# Examining Multilevel Influences on Depressive Symptoms Among Korean Older Adults: The Interplay of Individual and Regional Factors

**DOI:** 10.3390/healthcare13080870

**Published:** 2025-04-10

**Authors:** Miri Kim, Soondool Chung

**Affiliations:** 1 Department of Social Welfare, Korea National University of Transportation, Chungju 27469, Republic of Korea; mirikim@ut.ac.kr; 2 Department of Social Welfare, Ewha Womans University, Seoul 03760, Republic of Korea

**Keywords:** mental health, depressive symptoms, social isolation, living environment, age-friendly environment, multilevel model, older adult, South Korea

## Abstract

**Background/Objectives**: This study investigates how individual- and regional-level factors jointly influence depressive symptoms among older adults in South Korea, a rapidly aging society facing growing mental health concerns. **Methods**: Multilevel structural equation modelling with Monte Carlo confidence interval testing is used to analyze a cross-sectional, secondary dataset of 600 Korean older adults aged 65 years. The data come from the 2020 Ewha Study of Intergenerational Issues (ESoII), which is collected using multistage-quota sampling by age, gender, and region to ensure population representativeness across 14 cities and provinces. **Results**: Significant direct and indirect effects are observed at both individual and regional levels. At the individual level, aging anxiety is associated with social isolation (β = 0.208, *p* > 001) and depressive symptoms (β = 0.224, *p* < 0.001); social isolation is also associated with depressive symptoms (β = 0.288, *p* > 0.001), and mediates the relationship between aging anxiety and depression (95% CI = [0.016, 0.065]). At the regional level, age-friendly environments are associated with social isolation (β = −0.287, *p* < 0.05), which in turn is associated with depressive symptoms (β = 0.403, *p* < 0.01). The mediation effect of social isolation in the relationship between age-friendly environments and depressive symptoms is statistically significant (95% CI = [−0.022, −0.004]). **Conclusions**: The findings suggest that social isolation is a key mechanism linking both psychological and environmental risk factors to depression in later life. Promoting age-friendly environments may be an effective strategy for reducing social isolation and improving mental health outcomes among older adults. Interventions should consider both individual vulnerabilities and structural supports.

## 1. Introduction

With population aging and increasing life expectancy, maintaining a healthy life in old age has become a significant concern for individuals and society as a whole. South Korea, being the fastest-aging nation in the world, faces unprecedented challenges in this regard. The proportion of the older population in Korea is projected to rise from 19.2% in 2024 to 40.1% in 2050, an unprecedented increase [1]. Meanwhile, the lack of mental health care for increasing numbers of older adults in Korea has become a significant challenge. Korean older adults have consistently shown the highest rates of suicide and poverty among the Organization for Economic Co-operation and Development (OECD) countries, and a substantial number of them experience significant depressive symptoms. Specifically, 13.5% of older adults in Korea exhibited such symptoms, compared to the global average of 5.7% [2,3]. These statistics underscore the urgent need to address depression among older Korean adults as a significant mental health issue in later life.

Depression in later life is shaped by complex biological, psychological, and social mechanisms. Recently, researchers have emphasized the importance of environmental factors, especially for older adults who are more likely to remain within their local surroundings. Inadequate neighborhood conditions—such as poor infrastructure, lack of services, and weak social ties—have been linked to increased psychological distress and depressive symptoms [4,5,6]. Therefore, to more fully understand the mental health of older adults, scholars have called for approaches that integrate not only individual-level characteristics but also the broader regional environment in which they live [7].

The age-friendly environment (AFE) framework proposed by the World Health Organization (WHO) provides a multidimensional lens to address these concerns. AFEs promote active aging and aging-in-place by supporting physical, social, and service environments tailored to the needs of older people [8]. Prior research indicates that these components of AFEs are associated with lower levels of social isolation, reduced aging anxiety, and improved psychological well-being [9,10]. However, most existing studies have focused separately on either individual-level factors or environmental-level attributes, overlooking the complex ways in which they interact to influence late-life depression. Additionally, few studies have adopted a multilevel analytical approach to examine these cross-level dynamics.

Therefore, this study aims to examine how individual psychological factors and regional-level age-friendly environments interact to influence depressive symptoms among older adults in Korea. By integrating multilevel data and exploring mediation pathways, this research addresses a key gap in the literature and provides practical implications for designing effective policy interventions. The findings of this study can serve as a guideline for developing macro-level measures to reduce depressive symptoms, alleviate social isolation, and decrease aging anxiety among older adults, ultimately creating a sustainable and supportive environment for older people’s well-being.

## 2. Materials and Methods

### 2.1. Data and Sample

The study analyzed 600 older adults aged 65 and older from a cross-sectional, secondary dataset from 2020 Ewha Study of Intergenerational Issues (ESoII), conducted by the Ewha Institute for Age Integration Research of South Korea. A multistage-quota sampling strategy was employed based on age, gender, and geographical areas to ensure representativeness across seven metropolitan cities and eight provincial cities in South Korea. A total of 89 municipal-level districts (si, gun, and gu) were randomly selected from 226 districts, proportionally based on the population distribution within each of the 14 provincial-level divisions. Data collection took place through face-to-face interviews conducted by trained researchers between 17 June and 31 July 2020. The interviews were conducted in settings selected to ensure participants’ comfort and accessibility, such as community welfare centers and participants’ homes. The interviewers were instructed to check responses in real time to prevent missing values. This study received ethical clearance from an institutional ethics committee (IRB Number: 202005-0003-01), and informed consent from participants has been appropriately obtained.

### 2.2. Assessment of Depressive Symptoms

Depressive symptoms were assessed using the ten-item Center for Epidemiologic Studies Depression Scale (CES-D-10) [11]. The scale was translated and validated for use in Korea by Shin [12]. Participants rated the frequency of experiencing depressive symptoms over the past week on a four-point Likert scale (0 = rarely or none of the time; 3 = most of the time). Total scores range from 0 to 30, with higher scores indicating greater depressive mood. A score above 16 suggests the presence of significant depressive symptoms. The internal consistency of this scale, measured by Cronbach’s alpha, was 0.813.

### 2.3. Assessment of Age-Friendly Environment

The age-friendliness of the administrative districts of si-gun-gu (cities, counties, and districts) was assessed using the Age-friendly City Index developed by Ko et al. [13]. This index encompasses dimensions of the physical, social, and service environments, aligning with the guidelines set forth by the World Health Organization (WHO) for age-friendly cities. According to the WHO, these three domains are essential components that shape older adults’ ability to participate safely, comfortably, and actively in their communities [8,13].

To facilitate comparison, the original values of each indicator were standardized using *t*-scores, as the indicators varied in value ranges, units, and characteristics. Outliers were handled by substituting them with the nearest data point, and item-level confirmatory factor analysis (CFA) was conducted to eliminate items with negative factor loadings and factor loadings below 0.3. Following the data cleaning process, the final indices for each dimension were as follows.

First, the physical environment dimension included the number of security lights, heat wave shelters, public toilets, and road crossing safety scores (measured by traffic accidents involving older populations) relative to the number of older populations in each district. Data for these indicators were retrieved from the Open Government Data Portal of the Ministry of the Interior and Safety, the National Disaster and Safety Portal of the Ministry of the Interior and Safety, the Local Administration Integrated Information System of the Ministry of the Interior and Safety, and the Traffic Accident Analysis System of the Road Traffic Authority, respectively.

Second, the social environment dimension included the number of leisure welfare facilities for older adults, traditional markets, and organizations providing employment programs for older adults, relative to the number of older populations in each district. Data for these indicators were obtained from e-country indicators of Statistics Korea, the Open Government Data Portal of the Ministry of Interior and Safety, and Senior Job Yeogi of the Ministry of Health and Welfare, respectively.

Third, the service environment dimension included the number of health welfare facilities for older adults, in-home welfare facilities for older adults, and care service facilities for older adults, relative to the number of older populations in each district. Data for the first two indicators were retrieved from e-country indicators of Statistics Korea, while data for the latter indicator were obtained from the Open Government Data Portal of the Ministry of the Interior and Safety. The scores from each dimension were aggregated to generate the AFE score for each district. Higher scores indicate a higher level of age-friendliness within the district.

### 2.4. Assessment of Social Isolation

Social isolation was assessed using five items from the Emotional/Social Aspects of Loneliness and Isolation (ESLI) scale [14], validated in Korean by Lee [15]. Five items from the ESLI scale were selected, which specifically assess the objective state of lacking intimacy and attachment in one’s social network. Responses were rated on a five-point Likert scale (1 = not at all; 5 = very much). Higher scores reflect greater social isolation. Internal consistency was high (Cronbach’s alpha = 0.892).

### 2.5. Assessment of Aging Anxiety

Aging anxiety was measured using a 17-item Korean aging anxiety scale developed and validated by Jung [16]. Ten items on the scale measure psychosocial anxiety, whereas seven items measure financial anxiety. The items include concerns such as loneliness, relocation in later life, and financial insecurity. Higher scores indicate higher levels of aging anxiety. The scale showed high internal consistency (Cronbach’s alpha = 0.901).

### 2.6. Control Variables

The study’s control variables included gender (1 = male, 0 = female), spouse status (1 = yes, 0 = no), self-rated physical health status (1 = bad, 2 = okay, 3 = good), and monthly income (1 = below 1,000,000 won; 2 = below 2,000,000 won; 3 = below 3,000,000 won; 4 = below 4,000,000 won; 5 = above 4,000,000 won). In the within-level model, control variables were included as covariates and were linked to aging anxiety, social isolation, and depressive symptoms.

### 2.7. Data Analysis

This study employed the multilevel structural equation modeling (MSEM) method as the analytical approach, which combines the features of multilevel modeling (MLM) and structural equation modeling (SEM) to investigate complex structural relationships among variables at different levels [17]. Data analysis was conducted using SPSS 18.0 and Mplus 7.0. Initially, a descriptive analysis was performed to provide an overview of the data. The normal distribution assumption was assessed using criteria such as skewness values between −2 and +2 and kurtosis values between −7 and +7 [18]. Pearson correlations were calculated to examine linear associations between research variables and to identify potential multicollinearity. The intraclass correlation coefficient (ICC) was also assessed to examine the multilevel structure of the dataset [19]. ICC values ranging from 0.05 to 0.09 indicate a low effect, values from 0.10 to 0.14 indicate a moderate effect, and values above 0.15 indicate a large effect [20]. Next, item parceling was applied to address estimation difficulties arising from non-normality and coarse categorization in the data [21,22]. Parceling involves combining scores to create indicators with improved reliability, a reduced number of parameter estimates, increased model fit, and reduced sources of sampling error [23]. Parceling was particularly appropriate in this study given the complex multilevel SEM framework, which benefits from more parsimonious models and stable parameter estimates. For individual-level items, a balancing approach was used, while facet-representative parceling was applied to regional-level items.

A multilevel confirmatory factor analysis (MCFA) was conducted to simultaneously consider both levels of data and estimate within- and between-cluster effects. Latent factor scales were identified at each level by fixing the factor loading of the first item of each parcel to 1.0, with the remaining factor loadings, latent factor variances, and residual variances freely estimated. Residual standard deviations between levels were also estimated to avoid negative residuals. Model fit was evaluated using commonly accepted criteria (CFI ≥ 0.95, SRMR ≤ 0.08, RMSEA ≤ 0.08), which are widely used to ensure the scalability and interpretability of structural equation models [24,25]. The chi-square value was not considered due to its sensitivity to large sample sizes [26]. Furthermore, associations between level-1 and level-2 variables were examined using MSEM, with the variances of level-1 and level-2 variables separated. Centering was not performed since explicit centering is not required when testing general MSEM on raw data [17]. Lastly, a Monte Carlo simulation was conducted to examine the mediating effects of variables, considering the multilevel context [27]. The significance of the specific indirect effect was determined at α = 0.05 by examining whether the 95% Confidence Interval (CI) excluded zero [28].

## 3. Results

### 3.1. Characteristics of the Study Participants

Table 1 shows the socio-demographic characteristics of study participants. There were more females (55.67%) than males (44.33%), and the average age of participants was 73.77 years (SD = 5.80), ranging from 65 to 89. A total of 49.33% of respondents had an elementary school education, whereas 4.50% had a university education. Most participants were with a spouse (62.33%), and 27.83% of the respondents perceived their health status as bad. Regarding occupational status, 40.50% of the respondents still worked. Most participants received less than KRW 1,000,000 or USD 715 per month (31.00%).

Table 2 presents the descriptive statistics for the individual-level variables. The majority of participants exhibited low to moderate levels of depressive symptoms, with a mean value of 7.47 (SD = 4.33). Social isolation was reported at a moderate level, with a mean value of 2.20 (SD = 0.76). Conversely, aging anxiety was found to be moderate to high, with a mean value of 3.06 (SD = 0.61). Normal data distribution was assumed for all variables, as indicated by the highest absolute skewness value of 0.623 and the highest absolute kurtosis value of 0.553 [18].

The intraclass correlation coefficients (ICC) were examined to assess the multilevel structure of the data. The ICC values for depressive symptoms, social isolation, and aging anxiety were 0.241, 0.180, and 0.366, respectively. These values indicate a substantial level of between-cluster variation and support the use of multilevel analysis [20].

### 3.2. Characteristics of the Community

On average, 17.3% of the total population was comprised of older adults. The number of study participants in each region varied, ranging from a minimum of two participants to a maximum of 16 participants in a single district. The average number of participants per district was seven. In terms of the AFE scores for the physical, social, and service domains, the mean value of the total AFE score was 150 (SD = 17.6). The district with the lowest AFE score had a mean score of 124.3, while the district with the highest AFE score had a mean score of 193.8 out of a maximum score of 300. Furthermore, significant correlations were found between depressive symptoms and the other three latent variables (physical, social, and service environments). However, there were no issues of multicollinearity, as the highest correlation value observed was 0.372.

### 3.3. Results for the Multilevel CFA

Table 3 shows the results for the MCFA—model fit was good for all of the indices (RMSEA = 0.015, CFI = 0.996, SRMR (within) = 0.026, and SRMR (between) = 0.067). In the within-level and between-level models, standardized factor loadings fell within the acceptable range between 0.3 and 0.9 [29]. All factor loadings were significant at the 0.001 level.

### 3.4. Results for the Multilevel SEM

Table 4 presents the results of the multilevel structural equation modeling (MSEM). The final MSEM model demonstrated a good fit (RMSEA = 0.016, CFI = 0.994, SRMR (within) = 0.026, SRMR (between) = 0.058). At the within-level, significant direct effects were found. Aging anxiety had a positive and significant effect on social isolation (*β* = 0.208, *p* < 0.001), social isolation had a positive and significant effect on depressive symptoms (*β* = 0.288, *p* < 0.001), and aging anxiety had a positive and significant effect on depressive symptoms (*β* = 0.224, *p* < 0.001). Among the control variables, gender did not show significant associations with any variables. However, spouse status was negatively correlated with depressive symptoms (*β* = −0.168, *p* < 0.001). Monthly income exhibited a negative association with aging anxiety (*β* = −0.212, *p* < 0.001) and social isolation (*β* = −0.178, *p* < 0.05). Furthermore, self-rated physical health status was negatively correlated with aging anxiety (*β* = −0.130, *p* < 0.05) and depressive symptoms (*β* = −0.211, *p* < 0.001). At the between-level, significant direct effects were also observed. The AFE had a negative and significant effect on social isolation (*β* = −0.287, *p* < 0.05), and social isolation had a positive and significant effect on depressive symptoms (*β* = 0.403, *p* < 0.01).

### 3.5. Results for the Indirect Effects

Results for the indirect effects are shown in Table 5. At the within-level, there was a statistically significant indirect effect of aging anxiety and social isolation on depressive symptoms, with a 95% confidence interval (CI) ranging from 0.016 to 0.065. At the between-level, there was a statistically significant indirect effect of an AFE and social isolation on depressive symptoms, with a 95% CI ranging from −0.022 to −0.004.

## 4. Discussion

This study examined the multilevel structural relationships between AFEs, aging anxiety, social isolation, and depressive symptoms in older adults, taking into account the multilevel structure and multidimensional nature of the environment. At the within-level analysis, the findings supported the notion that higher levels of social isolation were associated with increased depressive symptoms among older adults. This aligns with previous research emphasizing the significance of maintaining satisfactory social relationships for maintaining good mental health, particularly in later life [30]. Individuals with more social connections tend to have higher self-esteem, greater empathy, and more trusting and cooperative relationships. These factors contribute to a positive feedback loop of social, emotional, and physical well-being, ultimately leading to lower levels of depressive symptoms.

Indeed, governments worldwide have increasingly recognized the detrimental impact of social isolation and loneliness on their citizens, particularly with the heightened discussions prompted by the global COVID-19 pandemic. In 2018, the United Kingdom took a significant step by appointing its first Minister of Loneliness, and this effort was further amplified in response to the COVID-19 outbreak with the introduction of a comprehensive plan to address the exacerbated social isolation and loneliness [31]. Similarly, Japan appointed Japan’s first Minister of Loneliness in 2021 to implement measures aimed at preventing social isolation. In Korea, the Act on the Prevention and Management of Lonely Deaths was established in 2020 to address the emerging social issue of solitary deaths. It is widely acknowledged across these countries that addressing social isolation and loneliness requires collective efforts and collaboration from multiple stakeholders, including individuals, communities, organizations, schools, employers, and governments, since government action alone is deemed insufficient to effectively tackle this issue [32].

The present study’s findings regarding the association between higher aging anxiety and increased depressive symptoms among older adults are consistent with existing research that has demonstrated significant links between aging anxiety and depression [33,34]. In the context of Korea, societal changes, such as the rise of nuclear families, have contributed to transformations in traditional family structures. These changes have given rise to increased aging anxiety among older adults, as they question whether they will have adequate psychological and financial support in later life without the support network of their families [35]. Such concerns can have detrimental effects on their mental health and contribute to higher levels of depressive symptoms.

Lastly, the results of this study support the mediating role of social isolation in the relationship between aging anxiety and depressive symptoms at the within-level. Individuals with high levels of aging anxiety, characterized by concerns about health declines, changes in social relationships, and financial instability in later life, are less likely to engage in active aging, which is a significant factor associated with lower levels of depressive symptoms in older age [9]. Moreover, high levels of aging anxiety can hinder individuals’ engagement in physical activity and diminish their motivation for life [36]. Consequently, individuals expressing concerns about their later life are more prone to adopting a sedentary lifestyle and withdrawing from social groups, thereby increasing their risk of experiencing social isolation, which, in turn, can contribute to the development of depression [37].

At the between-level, this study examined the mediating effect of social isolation in the relationship between an AFE and depressive symptoms. The findings align with previous research that has demonstrated the direct influence of neighborhood characteristics on mental health and their capacity to modify the effects of other factors related to mental well-being, such as one’s social network composition [38,39]. The neighborhood serves as a fundamental context for maintaining existing social connections and fostering new ones, thus influencing the mental health of its residents [40,41]. Specifically, an AFE enhances social engagement opportunities for older adults and facilitates aging-in-place, thereby maximizing the maintenance of social networks even in old age. For example, the presence of local welfare centers, accessible gathering places, and community programs specifically designed for older adults can encourage regular interactions and reduce the risk of social withdrawal. These environmental features make it easier for older adults to remain socially connected, which, in turn, lowers their experience of loneliness and isolation. Reduced social isolation not only improves emotional well-being but also acts as a protective factor against depressive symptoms by fostering a sense of belonging and support.

These results suggest that policies aimed at building age-friendly environments could reduce mental health disparities in aging populations. However, to translate these findings into practice, local and national governments must consider integrated strategies that combine infrastructure improvements with programs that actively support social participation. For instance, Denmark has demonstrated how participatory urban design can foster age inclusivity. In a notable project in Copenhagen, city planners and researchers implemented a co-design process involving over 100 older residents from a low-income neighborhood in redesigning their local environment. Through collaborative workshops and full-scale prototyping, participants contributed ideas such as improved benches, safer pathways, and better gathering spaces. This process not only enhanced the built environment but also empowered seniors by valuing their lived experiences in decision-making [42]. Denmark’s broader commitment to universal design and age-inclusive public space illustrates how thoughtful urban planning can enhance active aging across generations. Adapting such participatory and integrative approaches to the Korean context may strengthen the effectiveness of AFE interventions.

Furthermore, in line with continuity theory, older adults strive for stability as they navigate the changes associated with aging. This involves maintaining continuity in their habits, associations, relationships, preferences, attitudes, and behaviors developed over a lifetime. Relocation and abrupt changes in lifestyle and social relationships can significantly impact the psychological well-being of older adults, as it disrupts their sense of continuity [43]. By residing in an AFE, older adults can preserve continuity in their lives, thereby enhancing their ability to adapt to the challenges associated with aging, including the potential deterioration of mental health. Actively participating in the community and expanding social circles within an AFE can help mitigate social isolation and promote better mental health outcomes for older adults [44].

However, contrary to expectations, the results of the current study did not support the mediation effect of aging anxiety in the relationship between AFEs and depressive symptoms at the regional level. This finding is inconsistent with previous studies that have demonstrated significant associations between AFE characteristics and lower levels of aging anxiety in older adults, which, in turn, have been linked to lower levels of depression [34,45]. One possible explanation for the lack of mediation effect in this study is that cities in Korea are still in the early stages of adopting the concept of an AFE, and the efforts implemented thus far may not have been sufficient to effectively change people’s perceptions and reduce aging anxiety. While the physical and social enhancements from age-friendly policies are evident, research suggests that psychological benefits for older adults may take longer to emerge [46]. For instance, making neighborhoods more walkable may not immediately reduce depressive symptoms or aging-related anxiety; such outcomes often require sustained engagement over time. Short-term health or lifestyle interventions tend to have limited initial effects on individual well-being, as improvements in mental health typically depend on gradual, multifaceted changes in behavior and environment [46]. Therefore, future evaluations should take into account both the stage of AFE implementation and the level of community awareness when assessing their impact.

Furthermore, the inconsistency in the results could also be attributed to measurement differences. For example, previous studies such as Lee and Park [47] did not incorporate the financial aspects of aging anxiety in their analysis. However, investigating anxiety related to financial factors is particularly important in the Korean context, as a significant proportion of older Korean adults experience poverty [48]. The inclusion of financial concerns in future research may provide a more comprehensive understanding of the relationship between AFEs, aging anxiety, and depressive symptoms among Korean older adults.

Lastly, the current study did not find significant mediation effects of aging anxiety and social isolation in the relationship between an AFE and depressive symptoms at the regional level. This result suggests that living in an AFE may not have a substantial influence on reducing the aging anxiety of older adults, despite the significant association observed between aging anxiety and social isolation at the within level. The lack of mediation effects at the regional level may indicate that the specific features of the AFE examined in this study were not sufficient to effectively alleviate aging anxiety and subsequently reduce social isolation and depressive symptoms among older adults. It is possible that additional factors or interventions may be needed to address the complex interplay between aging anxiety, social isolation, and depressive symptoms at the regional level.

This study has revealed a significant association between AFEs and the level of social isolation and depressive symptoms among older adults. Based on these findings, we propose effective measures for adopting AFE factors to reduce social isolation and depressive symptoms among older adults.

First, it is important to develop the physical infrastructure of AFEs to maximize older adults’ engagement in daily activities. Difficulties in performing everyday tasks can result in a loss of independence and functional decline, particularly when older adults confine themselves to their homes. Therefore, creating an environment that enables older adults to move around comfortably and safely is crucial. For instance, local governments in Korea have implemented measures such as the designation and operation of heat wave shelters to protect older adults from extreme heat. These shelters not only provide a safe space but also encourage information exchange and social connections within the older adult community, enhancing their overall well-being and reducing social isolation.

Second, the level of age integration in communities should be enhanced as part of the journey towards creating age-friendly cities. An age-integrated society encourages people of all ages to actively participate in a wide range of activities throughout their lives. By promoting the engagement of older adults in areas such as education, politics, social activities, and sports, and facilitating interactions with individuals from different age groups, we can foster mutual understanding, reduce ageism, and cultivate positive perceptions of aging. Creating opportunities for intergenerational connections and collaboration can lead to a more inclusive and supportive community for older adults.

Third, researchers and governments should maintain continuous and active efforts at the national level to promote AFEs to effectively respond to the rapid urbanization and population aging. Given the accelerated pace of population aging in Korea, there is an urgent need to develop a comprehensive national-level policy that addresses the specific needs of older adults and ensures the country’s sustainability. Embracing a proactive approach at the national level enables cities and regions to adequately prepare for the social, economic, and healthcare implications of population aging. This involves addressing regional disparities in resources and services, fostering age-friendly practices, and establishing a legal and institutional framework to support the development of AFEs. Such endeavors are crucial for creating sustainable and inclusive communities that cater to the evolving needs of older adults and foster their overall well-being. Through the provision of sufficient support and infrastructure, the potential of the aging population can be maximized, leading to enhanced long-term sustainability for the country.

There are several limitations to this study that should be acknowledged. First, the use of cross-sectional data limits our ability to understand how individual and regional factors influence depressive symptoms over time. Longitudinal research is needed to establish causal relationships and clarify the temporal sequence of these associations. This would provide deeper insights into how these factors dynamically interact and evolve among older adults. Second, although the current study focused on objective indicators of AFEs, future research should consider older adults’ perceived age-friendliness of their living environment through qualitative methods such as interviews or focus groups. Third, while this study focused on the Korean context, cross-national comparisons with successful active and age-friendly aging policies in other countries could help contextualize the findings and identify transferable practices.

Despite the limitations, this study makes a significant contribution to our understanding of the intricate multilevel relationship between AFEs, social isolation, aging anxiety, and depressive symptoms in older adults. The findings of this study have implications that extend beyond the specific context of Korea and can be applied to other countries as well. The concept of AFEs is gaining global recognition, with many countries actively promoting policies and initiatives to create supportive and inclusive communities for older adults. The multilevel structural modeling approach used in this study, along with the focus on social isolation, aging anxiety, and depressive symptoms, provides a framework that can be adapted and applied in different cultural and geographical contexts.

For example, the study’s emphasis on developing AFEs to alleviate social isolation and depressive symptoms among older adults can provide valuable insights for other countries that are also experiencing significant population aging. By 2050, more than two-thirds of OECD countries are projected to have at least one-quarter of their population aged over 65 years [46]. The increasing old-age dependency ratios will exert significant pressure on the financing of pensions, healthcare, and long-term care, while also reducing the capacity of families to provide comprehensive care for older adults. Therefore, addressing the challenges posed by population aging and the increasing old-age dependency ratios requires a comprehensive and innovative approach, such as the development and implementation of AFEs. The adoption of age-friendly measures will facilitate the promotion of a dignified and fulfilling life for aging populations within the community and ensure a sustainable and inclusive approach to aging societies.

## 5. Conclusions

In conclusion, this study highlights the multilevel influence of both individual psychological vulnerabilities and regional environments on depressive symptoms among older adults in South Korea. Specifically, aging anxiety and social isolation at the individual level, as well as age-friendly environmental characteristics at the regional level, were found to significantly impact mental health outcomes. These findings reinforce the importance of designing integrated policy and community-level interventions that both reduce social isolation and promote supportive physical and social environments, which efforts can contribute to a healthier, more inclusive aging process. Future research should build on these findings by employing longitudinal designs to assess the long-term impact of age-friendly environments on depression and social integration among older adults.

## Figures and Tables

**Table 1:** Socio-demographic characteristics of study participants (*N* = 600).

		Frequency	%
Gender	Female	334	55.67
	Male	266	44.33
Age		M = 73.77, SD = 5.80 (Range: 65–84)	
Educational attainment	Elementary school education	296	49.33
	Middle school education	127	21.17
	High school education	150	25.00
	University education	27	4.50
Spouse status	Living alone	226	37.67
	Living with spouse	374	62.33
Self-rated physical health status	Bad	167	27.83
	Okay	212	35.33
	Good	221	36.83
Employment status	Not working	357	59.50
	Working	243	40.50
Monthly income	Below KRW 1,000,000 (Below USD 715)	186	31.00
	KRW 1,000,000~2,000,000 (USD 715~1430)	156	26.00
	KRW 2,000,000~3,000,000 (USD 1430~2145)	130	21.67
	KRW 3,000,000~4,000,000 (USD 2145~2860)	59	9.83
	Above KRW 4,000,000 (Above USD 2860)	69	11.50

Note. USD values are approximate, based on an exchange rate of USD 1 ≈ KRW 1400.

**Table 2:** Descriptive statistics for individual-level variables.

	Mean (SD)	Range	Min–Max	Skewness	Kurtosis	ICC
Depressive symptoms	7.47 (4.33)	0–30	0–23	0.62 (0.10)	0.10 (0.20)	0.24
Social isolation	2.20 (0.76)	1–5	1.00–4.40	0.47 (0.10)	−0.16 (0.20)	0.18
Aging anxiety	3.06 (0.61)	1–5	1.47–4.76	0.01 (0.10)	−0.55 (0.20)	0.37

**Table 3:** Results for the MCFA.

Measure	B	S.E.	β
Within-level			
A1	1.000	-	0.846
A2	0.865	0.046	0.808
A3	0.948	0.047	0.847
S1	1.000	-	0.681
S2	1.116	0.071	0.851
S3	1.114	0.080	0.893
D1	1.000	-	0.722
D2	1.134	0.092	0.760
D3	1.200	0.083	0.814
Between-level			
Phys	1.000	-	0.462
Soci	2.365	0.840	0.802
Serv	1.214	0.311	0.379
A1	1.000	-	0.848
A2	1.007	0.311	0.818
A3 ^a^	0.937	0.066	1.000
S1	1.000	-	0.808
S2	0.971	0.153	0.863
S3 ^a^	1.117	0.137	1.000
D1	1.000	-	0.842
D2	0.869	0.185	0.793
D3	0.553	0.137	0.604

Note. A: aging anxiety; S: social isolation; D: depressive symptoms; Phys: physical environment; Soci: social environment; Serv: service environment. RMSEA = 0.015, CFI = 0.996, SRMR (within) = 0.026, and SRMR (between) = 0.067. ^a^ At the between-level, residual standard deviations were estimated to avoid negative residuals.

**Table 4:** Results for the MSEM.

Pathway	B	S.E.	β
Within-level			
Social isolation → Depressive symptoms	0.148	0.031	0.288 ***
Aging anxiety → Depressive symptoms	0.147	0.032	0.224 ***
Aging anxiety → Social isolation	0.267	0.081	0.208 ***
Gender → Aging anxiety	−0.009	0.045	−0.010
Spouse → Aging anxiety	0.073	0.049	0.078
Income → Aging anxiety	−0.072	0.018	−0.212 ***
Health → Aging anxiety	−0.074	0.033	−0.130 *
Gender → Social isolation	0.046	0.055	0.039
Spouse → Social isolation	0.014	0.073	0.012
Income → Social isolation	−0.078	0.032	−0.178 *
Health → Social isolation	−0.068	0.046	−0.094
Gender → Depressive symptoms	−0.002	0.027	−0.003
Spouse → Depressive symptoms	−0.103	0.025	−0.168 ***
Income → Depressive symptoms	−0.104	0.012	−0.061
Health → Depressive symptoms	−0.078	0.023	−0.211 ***
Between-level			
Social isolation → Depressive symptoms	0.325	0.120	0.403 **
AFE → Depressive symptoms	−0.024	0.019	−0.218
Aging anxiety → Depressive symptoms	0.079	0.138	0.117
AFE → Social isolation	−0.039	0.018	−0.287 *
Aging anxiety → Social isolation	0.128	0.117	0.152
AFE → Aging anxiety	−0.023	0.024	−0.145

Note. * *p* < 0.05, ** *p* < 0.01, *** *p* < 0.001.

**Table 5:** Results for the indirect relationships.

Pathways	Point Estimate (S.E.)	95% CI
Within-level		
Aging anxiety → Social isolation → Depressive symptoms	0.039(0.012)	[0.016, 0.065]
Between-level		
AFE → Social isolation → Depressive symptoms	−0.013(0.005)	[−0.022, −0.004]
AFE → Aging anxiety → Depressive symptoms	−0.002(0.006)	[−0.016, 0.009]
AFE → Aging anxiety → Social isolation → Depressive symptoms	−0.001(0.002)	[−0.006, 0.002]

Note. Pathways are significant if 95% CI does not include zero.

## Data Availability

Dataset available on request from the authors.

## Abbreviations

The following abbreviations are used in this manuscript:

AFE	age-friendly environment
